# miRNA-205 Nanoformulation Sensitizes Prostate Cancer Cells to Chemotherapy

**DOI:** 10.3390/cancers10090289

**Published:** 2018-08-25

**Authors:** Prashanth K. B. Nagesh, Pallabita Chowdhury, Elham Hatami, Vijaya K. N. Boya, Vivek K. Kashyap, Sheema Khan, Bilal B. Hafeez, Subhash C. Chauhan, Meena Jaggi, Murali M. Yallapu

**Affiliations:** Department of Pharmaceutical Sciences and Center for Cancer Research, University of Tennessee Health Science Center, Memphis, TN 38163, USA; pbhusett@uthsc.edu (P.K.B.N.); pchowdhu@uthsc.edu (P.C.); ehatami@uthsc.edu (E.H.), drvijayboya@gmail.com (V.K.N.B.); vkashya1@uthsc.edu (V.K.K.); skhan14@uthsc.edu (S.K.), BHAFEEZ@uthsc.edu (B.B.H.); SCHAUHA1@uthsc.edu (S.C.C.); mjaggi@uthsc.edu (M.J.)

**Keywords:** miR-205, docetaxel, prostate cancer, chemosensitivity and EMT

## Abstract

The therapeutic application of microRNA(s) in the field of cancer has generated significant attention in research. Previous studies have shown that miR-205 negatively regulates prostate cancer cell proliferation, metastasis, and drug resistance. However, the delivery of miR-205 is an unmet clinical need. Thus, the development of a viable nanoparticle platform to deliver miR-205 is highly sought. A novel magnetic nanoparticle (MNP)-based nanoplatform composed of an iron oxide core with poly(ethyleneimine)-poly(ethylene glycol) layer(s) was developed. An optimized nanoplatform composition was confirmed by examining the binding profiles of MNPs with miR-205 using agarose gel and fluorescence methods. The novel formulation was applied to prostate cancer cells for evaluating cellular uptake, miR-205 delivery, and anticancer, antimetastasis, and chemosensitization potentials against docetaxel treatment. The improved uptake and efficacy of formulations were studied with confocal imaging, flow cytometry, proliferation, clonogenicity, Western blot, q-RT-PCR, and chemosensitization assays. Our findings demonstrated that the miR-205 nanoplatform induces significant apoptosis and enhancing chemotherapeutic effects in prostate cancer cells. Overall, these study results provide a strong proof-of-concept for a novel nonviral-based nanoparticle protocol for effective microRNA delivery to prostate cancer cells.

## 1. Introduction

Prostate cancer (PrCa) is the second leading cause of cancer-related deaths in men in the United States [1]. Chemotherapy is the most effective option at present to treat both advanced stage and metastatic prostate cancer. Docetaxel (Dtxl) was approved by the Food and Drug Administration (FDA) in 2004 as a chemotherapeutic modality to treat various metastatic cancers including prostate cancer. However, Dtxl resistance remains a major challenge in clinical oncology [2,3,4]. Therefore, alternative therapeutic modalities that target cancer cells are a suitable option. It has been reported recently that tumor suppressor microRNAs play a critical role in PrCa tumorigenesis and progression [5,6]. This opens avenues for potential targets in prostate cancer. Among many micro RNAs (miRNAs), miR-205 is consistently downregulated in prostate cancer [7,8,9,10,11] and breast cancer. A reduced expression of miR-205 in prostate cancer promotes epithelial-to-mesenchymal transition (EMT), which leads to Dtxl resistance [12]. This scientific evidence suggests that if miR-205 is re-introduced in prostate cancer cells or tumors, it can slow down disease progression.

Development of an efficient miRNA delivery system is a highly challenging task due to rapid degradation of miRNAs in serum conditions and low cellular internalization. Viral vector-based delivery of miRNA(s) is conventionally used due to efficient transduction; however, its clinical translation is often limited by immunological and toxicological side effects. Moreover, limitation in transgenic capacity size [13] and their expensive nature makes them a difficult choice. These circumstances lead to the conclusion that development of a nonviral nanoplatform to deliver miRNAs is highly required, including a nanoplatform for prostate cancer therapy [14]. Various cationic polymers and formulations based on linear, branched, and dendritic poly(ethyleneimine) polymers (PEIs) or poly-lysine can be complexed with small interfering RNA (siRNA)/miRNA for successful local or systemic therapeutic applications [15,16,17,18]. Recently, many cationic liposomal formulations (transfection agents) were developed for this purpose. All of these formulations are able to deliver payloads to cells in either a caveolae- or clathrin-dependent manner, and are subsequently released due to the proton sponge effect at the endosomal compartment [19]. Poly(ethyleneimine) is a versatile carrier with low immune response, and it easily forms interpolyelectrolyte complexes with RNAs, and these complexes have been widely tested transfection reagents. High cellular toxicity and poor targeting concerns limit their use for cancer therapeutics, and therefore they are not generally advised for human therapeutic applications. Therefore, our goal is to develop an effective delivery system based on our novel delivery platform, i.e., multilayer magnetic nanoparticles [20,21], for miR-205 replacement therapy for prostate cancer.

Various magnetic nanoparticle-based approaches allow delivery of loaded chemo- /biomacromolecular/photothermal therapeutics and the inducement of magnetic resonance imaging signals. Due to superior biocompatibility and ultra-small particle size, magnetic nanoparticles are highly suitable for a medical cargo regimen designed to navigate treatment towards tumor tissues by enhancing circulation, improving permeation and retention in tissues, and providing greater internalization in cancer cells. Therefore, in this work, we investigated the characteristic role of PEI and poly(ethylene glycol) (PEG) surface coating on magnetic nanoparticles for miR-205 delivery to prostate cancer cells. While PEI coating facilitates miR-205 binding, PEG polymer chains provide better stability for the formulation. A systematic characterization of binding for magnetic nanoparticle(s) (MNPs) with PEI and PEG, along with the identification of the active moiety for conjugation, together represent a rigorous study of a balanced combination of properties for transfection applications. Our data suggest that magnetic nanoparticles conjugated with the PEI-PEG (termed as MNP-PEI-PEG or MPEI-PEG) platform not only exhibit cell membrane-penetrating characteristics, but also provide a hemocompatible nonviral transfecting vector. This formulation is capable of binding with miR-205 and inducing anticancer potential in prostate cancer cells. In addition, this miR-205-containing formulation acts as a chemosensitizer for docetaxel therapy in prostate cancer cells.

## 2. Results

### 2.1. Preparation and Characterization of miR-205-MPEI-PEG (NPs) Formulations

**Figure 1:**
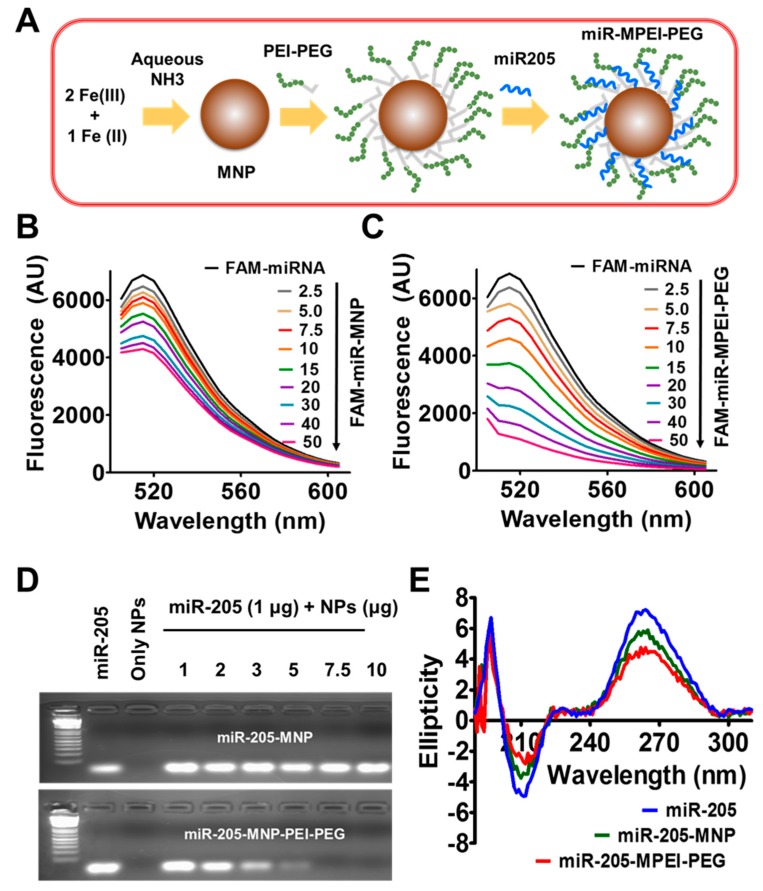
Generation of miR-205-NPs formulations (**A**) Preparative approach and hypothetical structure of miR-205 nanoformulations. PEI-PEG: gray line belongs to PEI and dotted greens belong to PEG. miR-205 binds to PEI. (**B**–**C**) Nanocomplexation assessment with miR-205. Fluorescence-based quenching study of fluorescein amidite (FAM)-labeled miRNA mimic MNP and MNP-MPEI-PEG (MPEI-PEG) nanoparticles. Note: MNP alone is also able to bind smaller amounts of miR-205, due to physical deposition on the nanoparticles but not due to binding. (**D**) Determination of nanocomplexation of miR-205 with MNP and MPEI-PEG through agarose gel electrophoresis. Data represent that 5 µg of nanocarrier is sufficient to hold 1 µg of miR-205 for delivery applications. (**E**) Evaluation of miR-205 binding with MNP and MPEI-PEG nanoparticles by circular dichroism (CD) spectral analysis.

A schematic illustration of magnetic nanoparticles (MNPs), magnetic nanoparticles conjugated with PEI-PEG (MPEI-PEG or simply NPs), and miR-205-MPEI-PEG (miR-205-NPs) formulations are shown in Figure 1A. PEI-PEG formed a multi-layered structure on the surface of the MNPs nanoformulation, stabilized through hydrophilic polymeric chains of poly(ethylene glycol) (PEG). The PEI in PEI-PEG polymeric chains exhibit complexation chemistry with miR-205, as shown previously in multiple studies. The hydrodynamic radius of the MNP and MNP-PEI-PEG (MPEG-PEI) formulation was 90 (PDI: 0.306) and 104.1 nm (PDI: 0.199), respectively (Figure S1A). After complexing miR-205 with MPEI-PEG, the particle size of the miR-205-MPEI-PEG formulation was increased to 117.2 nm (PDI: 0.246). This range of particle size (~100 nm) is considered optimal for retention within the tumor environment via an enhanced permeation and retention mechanism. The zeta potential of MNPs show −15.25 mV, while the MPEI-PEG formulation is obviously introducing positive zeta potential (17 mV) due to PEI layers in nanoformulations (Figure S1B). This positive zeta potential may support miR-205 binding on MPEI-PEG. Thus, it can be hypothesized that miR-205 could complex within its multi-layered structure. TEM results further confirmed a slight variation in the particle size and dispersive behavior of MPEI-PEG after miR-205 complexation, i.e., miR-205-NPs (Figure S1C).

### 2.2. miR-205 Efficiently Complexes with MPEI-PEG Formulation

The superior complexation capacity of MPEI-PEG formulations with miR-205 was confirmed employing three independent assays (Figure 1B–E). Complex/self-assembly formation between MPEI-PEG formulation (positive charge) and miR-205 (negative charge) occurs through an electrostatic binding interaction. This binding of miR-205 occurs on the PEI layers of the PEI-PEG polymer interface. A fluorescence quenching evaluation was employed to determine the complexation efficiency between the MNPs/MPEI-PEG formulations and fluorescein amidite (FAM)-labeled miRNA (the FAM label provides fluorescence) (Figure 1B–C). In the miR-205 and MNPs formulation quenching study, there is no significant decrease in fluorescence levels, indicating no efficient binding process occurred (Figure 1B). It is important to note that MNP alone is also able to bind smaller amounts of miR-205, which is due to physical deposition on the nanoparticles, not due to specific binding (Figure 1B). It was easy to observe a significant decrease in fluorescence levels of FAM-labeled miRNA upon titrating with MPEI-PEG (Figure 1C). The quenching rate is much higher with the addition of MPEI-PEG, which indicates that PEI plays a major role in the binding process. To examine the exact complexation ratio to achieve efficient binding between miR-205 and NPs, different compositions of MNPs/MPEI-PEG (0–10 µg) and miR-205 (1 µg) in nuclease-free water was vortexed and time allowed for binding (30 min). 5 µg of MPEI-PEG nanoformulation sufficiently bound almost all miRNA-205, which is indicated by the quenching of all miR-205 (no free miR-205 to show any fluorescence band); this is also indicated by a full retardation of the mobility of DNA in agarose gel. The MNP formulation that lacks PEI is unable to complex with miR-205, thus no retardation is observed, i.e., miRNA bands are highly visible (freely available miR-205), as with a free microRNA control (Figure 1D). These data indicate that PEI-PEG layers on MNPs are involved in complexation with miR-205, and at least a 1:5 wt % (miR-205: nanoparticle) ratio is optimum for efficient complexation.

A circular dichroism (CD) spectral study was also used to further confirm the miR-205 binding phenomenon with MNPs/MPEI-PEG nanoparticles (Figure 1E). The CD spectrum of pure miR-205 presents peaks at λ = 210 nm (negative), 260 nm (positive), and 300 nm (none or linear) (blue color). This represents the right-handed A-form characteristic of double-stranded RNA. MPEI-PEG formulation shows a similar effect on negative bands. A smaller band was observed in the positive excitation peak at λ = 260 nm corresponding to MPEI-PEG formulations (red color). This behavior is less than that observed with MNPs formulation (green color). All of these three independent binding studies confirm that MNP-PEI-PEG possesses a superior binding capacity with miR-205, and thus we chose this formulation (miR-205-NPs) for in vitro evaluation.

### 2.3. MPEI-PEG NPs Showed Superior Hemocompatibility

**Figure 2:**
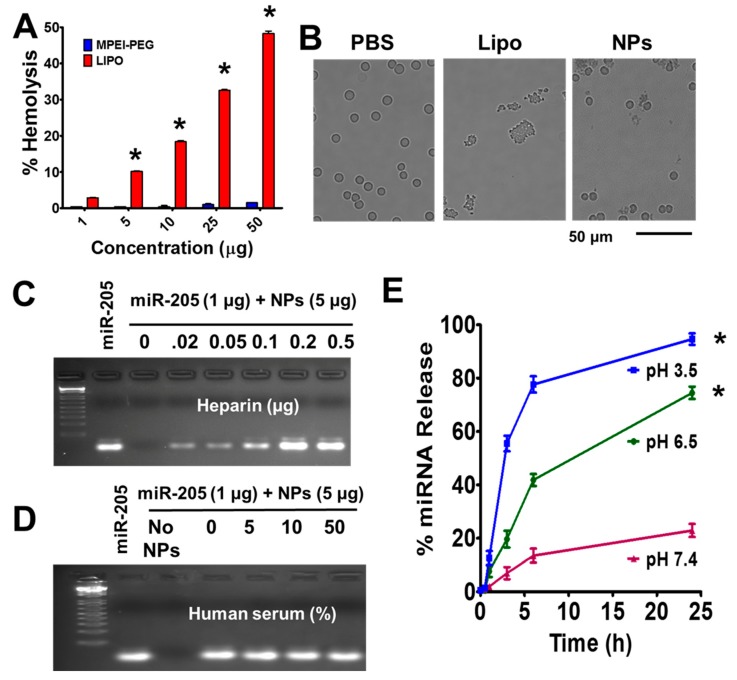
miR-205 delivery characteristics of miR-205-NPs. (**A**,**B**) Hemolytic activity of MNP-PEI-PEG nanocarrier. Brightfield microscopy at 20× magnification was employed for capturing hemolytic characteristic of human red blood cells. Scale bar: 50 µm. Note: NPs are not toxic but lipofectamine is at the tested concentrations. Data indicate that nanocarrier is hemocompatible, unlike lipofectamine. (**C**) Dissociation studies of miR-205 in presence of poly(anion) (heparin). (**D**) miRNA-205 stability in the presence of 0–50% Fetal Bovine Serum (FBS) concentration. Equal amount of each sample was incubated with 10 μL of FBS at 37 °C for 24 h prior to gel electrophoresis. Note: “No NPs” lane did not show any band due to the absence of miRNA. (**E**) FAM-miRNA release profile from the FAM-miR through fluorescence spectral analysis at variable pH solutions (7.4, 6.5 and 3.5). The significance level was * *p* < 0.05. Each experiment has been repeated three times.

A number of studies suggest that delivery systems can induce severe systemic toxicity. Thus, before choosing the MPEI-PEG (NPs) formulation as a delivery vehicle for miR-205, it was important to evaluate the hemocompatibility behavior and compare with a commercial formulation, Lipofectamine^®^. For this evaluation, an hemolysis assay was performed with freshly collected human red blood cells (RBCs). The hemolysis assay clearly shows that Lipofectamine^®^ induces some level of toxicity against RBCs [22] (Figure 2A, red bars). This effect varies directly with concentration, (1–50 µg/mL, 2.9–47% hemolysis). Our NPs formulation shows almost negligible toxicity, 5% hemolysis at the highest concentration (50 µg) (Figure 2A, blue bars). A positive control, sodium dodecyl sulfate (SDS) has severe toxicity (considered as 100%). In the morphological examination, the MPEI-PEG NPs formulation does not cause any changes in the membrane or whole cell morphology of RBCs and show very similar morphology of RBCs when those were treated with saline (control group) (Figure 2). In contrast, Lipofectamine carrier exhibits an aggregation of RBCs due to compromised membrane integrity (Figure 2B). These results clearly suggest the developed nanoformulation is hemocompatible and thus it can be used for delivery applications.

### 2.4. Fate of miR-205 in MPEI-PEG NPs Formulation

Heparin sulfate (0.025 µg), a polyanion, was able to dissociate miR-205 from the miR-205-MPEI-PEG formulation (Figure 2C) into the solution. Furthermore, there was a consistent increase of miR-205 release with increase in the heparin concentration ranging from 0.02–0.5 µg from the miR-205-MPEI-PEG nanoformulation. It is well known that microRNAs are unstable in serum [23]. To investigate the stability miR-205 in the miR-205-MPEI-PEG formulation in whole serum, the DNA agarose gel method was followed. miR-205 in serum incubation yielded no active miRNA in agarose gel (Figure 2D). The miR-205-MPEI-PEG formulation is protected from degradation due to tight binding of miR-205 at pH 7.4. To extract bound miR-205 from miR-205-MPEI-PEG, heparin sulfate (0.025 µg) was used. Subsequently, the extracted miR-205 was found to be equivalent as control in put miR-205 (without incubating in serum) (Figure 2D). All miR-205 was released from the formulation upon the addition of heparin sulfate, which plays a pivotal role in the displacement of whole miRNA into medium. The released free miR-205 from the miR-205-PEI-PEG nanoformulation in this manner results in DNA bands in agarose gel (2%) (Figure 2D). This clearly indicates that the released miRNA is intact and the band intensity profile is found to be about 95%, which is similar to naked miRNA (no serum incubation). Altogether, these results suggest that the miR-205-MPEI-PEG nanoformulation protects miRNA within the formulation from the degradation due to exonuclease activity of the serum. It is important to note that the ”No NPs” lane did not show any band due to the absence of miRNA.

Controlled release minimizes overlapping and interim effects on cancer cells. Here, we assessed the miRNA release profile at variable pH (3.5, 6.5, and 7.4) using FAM (fluorescent dye)-labeled miR (FAM-miR) with the help of a fluorescent spectra method. The spectra of samples were measured from 400–700 nm after incubation at variable time points (30 min to 24 h) and found λ_max_ at 530 nm. Results showed at pH 6.5 (tumor pH) a controlled release profile of FAM-miR while there was no/minimal release observed at pH 7.4. The release of FAM-miR was measured in terms of relative fluorescence units (RFU). We determined that there was minimal fluorescence at lower time incubations (30 min–1 h), while more release was observed at 3–6 h incubations (Figure 2E). The maximal and complete RFU was observed at 24 h incubation. However, minimal release of 20% was observed at pH 7.4, signifying that stability of the formulation and its acidic environment-based release. Altogether, results suggest that the prepared nanoformulation was triggered by pH-sensitive miRNA release from the formulation.

### 2.5. miR-205 is Efficiently Delivered and Preserved its Activity through NPs Formulation

**Figure 3:**
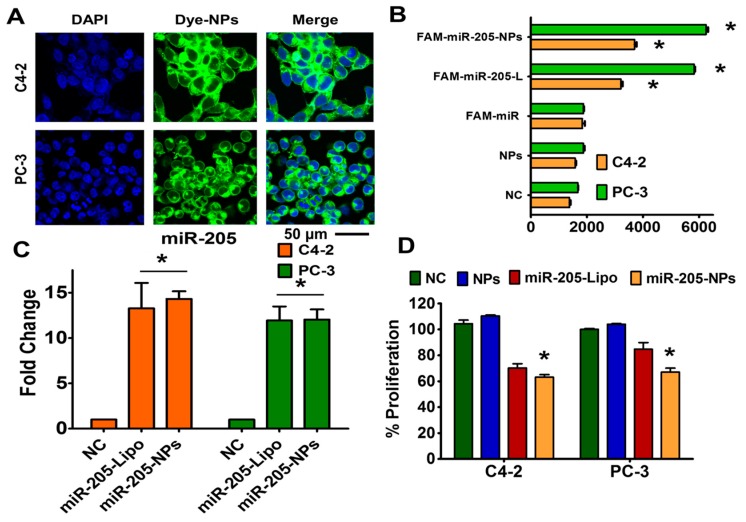
Cellular fate of miR-205-NPs formulation. (**A**) Cellular uptake of coumarin 6-labeled MPEI-PEG nanoformulation. Representative cellular internalized nanoparticles images were captured at 40× magnification using confocal microscopy. Green color arise from coumarin 6 in MPEI-PEG. Scale bar: 50 µm. (**B**) Quantitative measurement of FAM-miRNA mimic in cells treated with FAM-labeled miR-MPEI-PEG formulation. (**C**) Restoration of miR-205 through miR-205-NPs in PrCa cells. q-PCR gene expression studies reveals that treatments with miR-205-NPs reconstitutes the gene expression of miR-205. (**D**) miR-205-NPs influence cell growth in PrCa cells. Proliferation assays after miR-205 and miR-205-NPs transfection treatments in C4-2 and PC-3 cell lines using 3-(4,5-dimethylthiazol-2-yl)-5-(3-carboxymethoxyphenyl)-2-(4-sulfophenyl)-2H-tetrazolium, inner salt (MTS) assays. The significance level was * *p* < 0.05 with respect to NC/NPs. Each individual experiment has been repeated three times.

We performed cellular uptake in C4-2 and PC-3 cells to determine the penetrating capability of the MPEI-PEG formulation. Cellular uptake of coumarin 6 (green dye) through MPEI-PEG was evaluated by flow cytometry and confocal microscopy. The confocal images of coumarin 6-loaded and MPEI-PEG formulation-treated cells revealed a significant accumulation of nanoparticles located in the cytoplasm (Figure 3A). The extent of accumulation was concentration-dependent. Further, flow cytometry data demonstrates that uptake by MPEI-PEG was directly proportional to concentration, further demonstrating successful cellular uptake (Figure S2). In addition, we performed the cellular uptake studies of MPEI-PEG encapsulated using FAM-labeled (6-Carboxyfluorescein) Pre-miR Negative Control #1 (FAM-miR) (Thermo Fisher, Waltham, MA, USA) through flow cytometric studies. The transfection ability of our formulation was compared with a chemo agent (Lipofectamine^®^ 2000) (positive reference control). The results revealed that there was a consistent increase in the mean fluorescence intensity (MFI) of the cells after 24 h of treatment in both the lipofectamine (Lipo or L)-based transfection and the NPs formulation-based treatments (Figure 3B). Intracellular presence of FAM-miR-205-NPs facilitates the increase of MFI as in the lipofectamine transfection method.

A successful cellular uptake and active delivery of miR-205 through nanoformulation may induce anti-proliferative effects in prostate cancer cells. To study this phenomenon, we first evaluated the miR-205 endogenous expression in prostate cancer cells through miR-205 lipofectamine and miR-205-NPs-based transfection. The lipofectamine and miR-205-NPs delivery methods significantly upregulated endogenous miR-205 levels in C4-2 and PC-3 cells, respectively (Figure 3C). These results confirm that miR-205 delivery through MPEI-PEG is as effective as the commercial formulation. This indicates that miR-205 is active, which was confirmed by proliferation assay (Figure 3D). miR-205-NPs exhibited cellular growth inhibition beyond that of miR-205 lipofectamine-based delivery. Cells treated with miR-205 (no carrier or lipofectamine) were not affected by cellular inhibition. This confirms that miR-205 needs an efficient nanocarrier for efficient delivery into cancer cells. All these data suggest that miR-205 is highly active when encapsulated in NPs formulation. Therefore, the miR-205-NPs nanoformulation was chosen to conduct all other in vitro studies.

### 2.6. miR-205-NPs Formulation Induces Chemosensitization in Prostate Cancer Cells

Many studies demonstrate that prostate cancer cell lines and tumor cells exhibit suppressed miR-205 levels [24]. miR-205 loss is highly associated with tumor growth, invasion, and migration of cancer cells [25]. The reconstitution of miR-205 effectively inhibits proliferation of prostate cancer cells (Figure 3D) [26]. miR-205 restoration did not completely inhibit cancer cell survival but retarded its growth. It is imperative that complete annihilation of cancer cells is necessary at the clinic level [27]. To achieve profound clinical benefits, the chemosensitizing abilities of miR-205 is crucial for standard chemotherapies [28].

**Figure 4:**
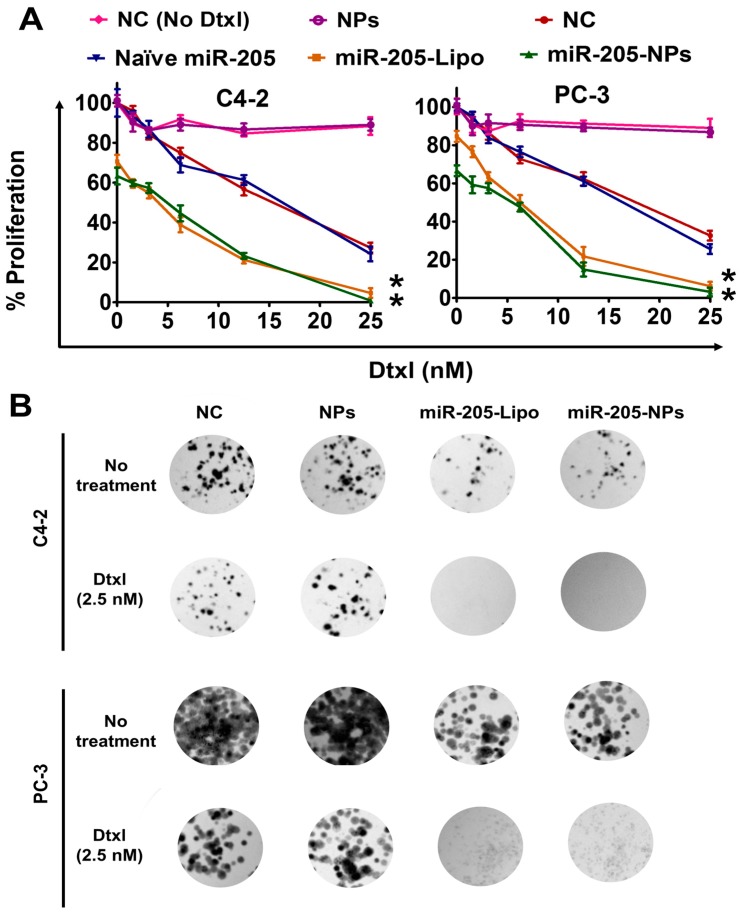
miR-205-NPs treatment chemosensitizes PrCa cells towards docetaxel therapy. (**A**) NC (No Dtxl), NPs, NC (non-targeting control), miR-205 naïve, miR-205-Lipo, and miR-205-NPs-treated PrCa cells (5 × 10^3^/well in 96-well plate) were treated with 0–25 nM Dtxl or respective control for 48 h and proliferation of cells was assessed by MTS assay. (**B**) NC, NPs, miR-205-Lipo, and miR-205-NPs-treated PrCa cells with 2.5 nM Dtxl. On day 14, cells were PBS-rinsed and stained with hematoxylin. Photographs of clonogenic pattern (in Multimage™ light cabinet) represent inhibition of clonogenic formation with miR-205-NPs formulation. Note: Figure 4B quantification was provided in Figure S3.

After confirming the successful restoration of miR-205 through NPs formulation in C4-2 and PC-3 cells, we directed our study towards the antiproliferative and chemo-sensitization effects of miR-205 using MTS and clonogenic assays. In the MTS assay, cell proliferation was remarkably inhibited with varying Dtxl treatment in miR 205-Lipofectamine (miR-205-Lipo) and miR-205-NPs transfected cells compared to Dtxl or miR-205 lipofectamine/miR-205-NPs formulation alone (no Dtxl treatment). This indicates that the combined cytotoxic effects of miR-205-L, miR-205-NPs, and Dtxl (5 nM) show synergism over Dtxl treatment. Cells treated with miR-205-Lipo and Dtxl or miR-205-NPs show reduced proliferation in C4-2 and PC-3 cells, respectively (Figure 4A), which is very significant compared to Dtxl treatment alone. Results of cell proliferation with varying Dtxl doses (0–25 nM) revealed that miR-205-expressing cells are more sensitive towards Dtxl treatment for 24 h. Additionally, chemosensitization of Dtxl was more effective in cells treated with miR-205-NPs and miR-205-Lipo, showing significant reduction in colony formation over less/null miR 205-expressing cells (Figure 4B). The order of reducing colony efficiently was NC miR-205-NPs ≥ miR-205-Lipo ≥ NC-Dtxl miR-205-Lipo + Dtxl miR-205-NPs + Dtxl (Figure 4A–B). For a better representation percent colonies were determined and presented in Figure S3. Overall, these data suggest that the combination of miR-205-MPEI-PEG and Dtxl treatment is more efficient in killing prostate cancer cells than Dtxl treatment alone.

### 2.7. miR-205-NPs Induce Apoptotic Signaling during Docetaxel Treatment

**Figure 5:**
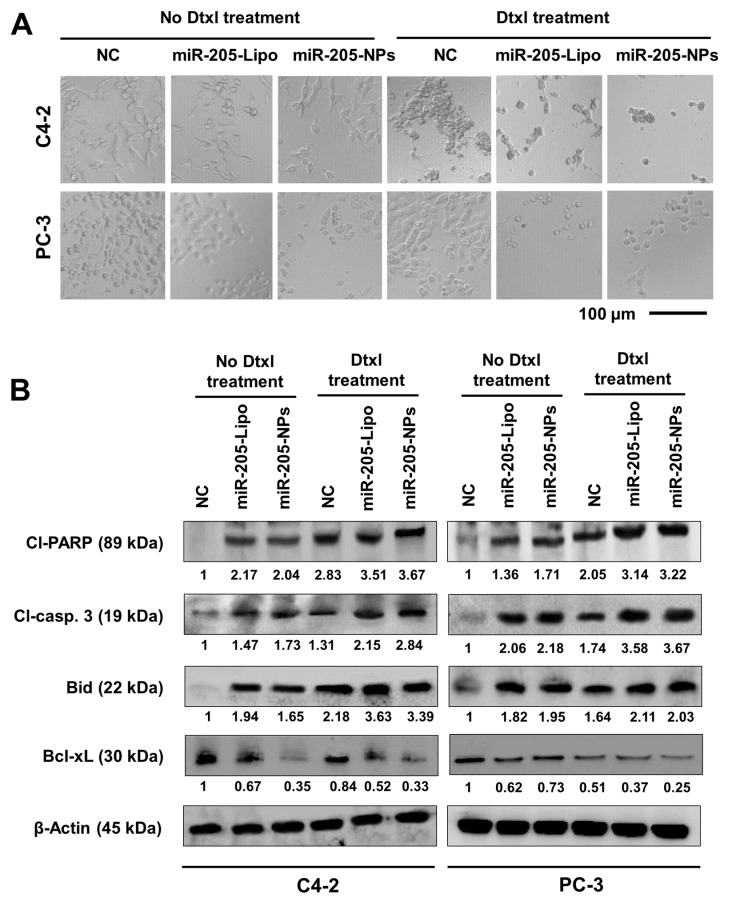
miR-205-NPs induce enhanced apoptotic potential of docetaxel in PrCa cells. (**A**) Representative microscopic images of various treatment groups after 24 h. Images were captured at 20× magnification. Scale bar: 100 µm. (**B**) Western blot analysis confirms the significant induction of apoptotic signaling proteins after the delivery of miR-205-NPs and Dtxl treatment. Cl-PARP and Cl-casp. 3 indicates cleaved PARP and cleaved caspase 3, respectively.

Morphological analysis was applied to visualize the effects of miR-205-NPs on C4-2 and PC-3 cells. The untreated control (NC) cells displayed a well-spread and flattened morphology (Figure 5A). miR-205 restoration through lipofectamine and miR-205-NPs in cells demonstrated reduced cell proliferation. The miR-205 restoration and Dtxl-combined treatment not only reduced the cell proliferation but showed pronounced apoptosis features. The apoptosis phenomenon is confirmed through cell rounding, reduced spreading, shrinkage, and retraction of cellular processes. This apoptosis signature was confirmed through Western blot analysis. A significant cleaved Poly (ADP-ribose) polymerase (PARP) and caspase 3 (apoptotic) and Bid (proapoptotic) proteins was observed in prostate cancer cells treated with either of miR-205-Lipo/miR-NPs and/or Dtxl as compared to control. In addition, a decrease in B-cell lymphoma-extra large (Bcl-xL) (pro-survival) protein expression levels was found with these treatments (Figure 5B). These results indicate that miR-205 restoration enhances Dtxl-induced apoptosis.

### 2.8. miR-205-NPs Formulation with Docetaxel Treatment Inhibits Metastatic Phenotype of PrCa Cells

**Figure 6:**
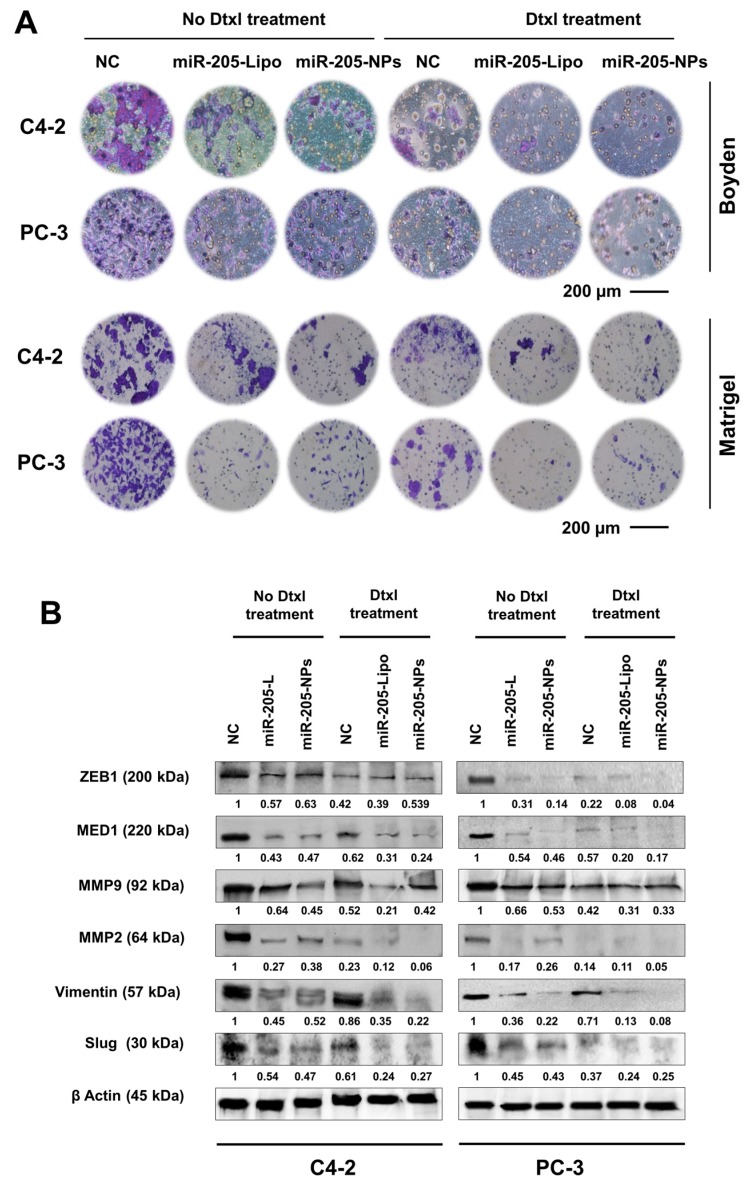
miR-205-NPs inhibits EMT signaling and sensitizes Dtxl treatment in PrCa cells. (**A**) miR-205-NPs treatment suppresses the migratory ability of C4-2 and PC-3 cells in presence/absence of the drug through Boyden chamber study. Images were captured at 20× magnification. Transwell assay with matrigel was performed to detect invasion activity of PrCa cells transfected with miR-205. Docetaxel treatments at 5 nM concentration. Scale bar: 200 µm. (**B**) Protein profiling studies of Control, miR-205 transfected and miR-205-NPs treated PrCa cells for 24 h for EMT signaling. Note: miR-205-L or miR-205-Lipo represents same and indicates to miR-205 transfected with lipofectamine.

To determine the effects of miR-205-NPs on metastatic attributes of prostate cancer cells, cell migration and invasion assays were performed using C4-2 and PC-3 cells. We investigated the effect of miR-205-NPs on the cellular motility and migration in prostate cancer cells using Boyden chamber assays (Figure 6A).

miR-205-NPs demonstrated a significant inhibition of the cell migratory potential of C4-2 and PC-3 cells in Boyden chamber assays, as compared to control (NC)-treated cells. Additionally, a matrigel invasion assay indicated 78 and 89% inhibitory invasiveness of C4-2 and PC-3 cells with miR-205-NPs treatment. Such inhibitory effect was further pronounced during Dtxl treatments (Figure 6A). The effect of miR-205-NPs on cell migration and cell invasion was also confirmed in real time PCR and Western blot studies. Downregulation of miR-205 in PrCa compared to normal tissues was previously reported and confirmed through miRNA expression profiling studies [29,30]. After restoration of miR-205, downstream targets like MED1 and ZEB1 were reduced with miR-205-lipo and miR-205-NPs treatments, and Dtxl treatments further reduced its expression (Figure 6B and Figure S4). As ZEB1 is the upstream regulator of EMT progression in constitutive gene expressing cells [31], we assessed the regulatory effects of protein expression of epithelial markers like E-cadherin, and mesenchymal markers like MMP9 and MMP2 in both of the cell lines. The expression of adhesion molecule E-cadherin is restored while the expression of MMP2, MMP9, Vimentin, and slug were inhibited upon re-expression of miR-205 (Figure 6B and Figure S4). Note: q–RT–PCR protocol and primers used in this expertiment was provided in Supplementary Materials as Table S1.

### 2.9. miR-205-NPs Exhibit Enhanced Intracellular Uptake of Rh 123 in PrCa Cells

Rhodamine 123 (Rh123), a member of the rhodamine family of fluorone dyes, has been used to examine membrane transport by the ABCB1 gene product, MDR1. MDR1 is viewed as the archetypal drug transport protein, and is able to efflux a large number of clinically relevant drugs [32]. All previous results described above confirm that miR-205-NPs formulation efficiently induces chemosensitization to Dtxl treatment.

**Figure 7:**
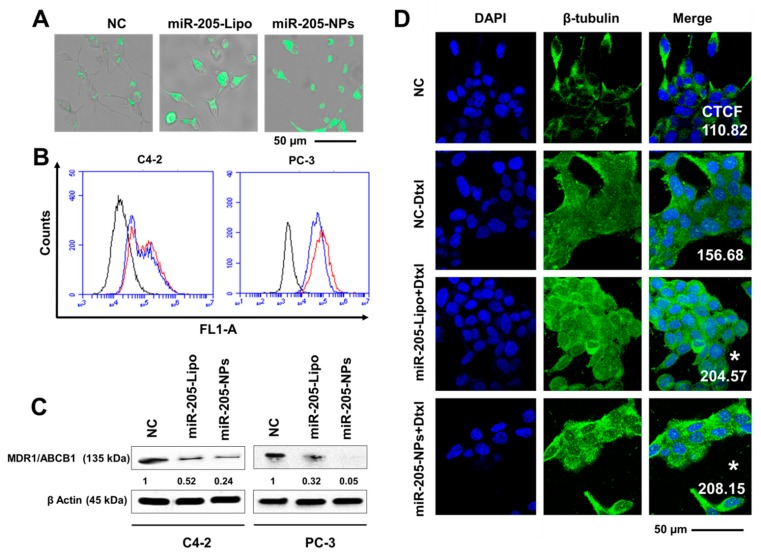
miR-205-NPs effectively inhibit Pgp activity and facilitates Dtxl chemosensitization. (**A**,**B**) Rh123 Dye exclusion studies were performed in cells through morphological and flow cytometric methods. miR-205-NPs formulation facilitates Rh123, demonstrating its chemosensitizing potential. (**C**) miR-205-NPs inhibited MDR1/ABCB1 expression in both C4-2 and PC-3 cells and densitometry studies were calculated. (**D**) miR-205-NPs promote microtubule stabilization in PrCa cells. Except for non-targeting control (NC), 5 nM Dtxl was used in all treatment groups. Treatment period was 8 h. Images were captured at 40× magnification using confocal microscopy. Scale bar: 50 µm. Corrected total cell fluorescence (CTCF) values were calculated using ImageJ and showed as insets. The level of significance was * *p* < 0.05. Each individual experiment has been repeated three times.

To prove such chemosensitization, an Rh123 uptake experiment was performed. These data show an increase in the fluorescence intensity of Rh123 in cells transfected with miR-205/miR-205-NPs (Figure 7A). This may be due to less dye efflux due to the inactivation of MDR1/ABC protein. These results were further evident from Rh123 fluorescence observed from flow cytometry studies (Figure 7B). A clear shift of cellular peak towards the right (from black to blue and red histograms) in miR-205/miR-205-NPs nanoformulation transfected cells in comparison to untreated controls.

### 2.10. miR-205-NPs Show Pronounced Tubulin Stabilization

**Figure 8:**
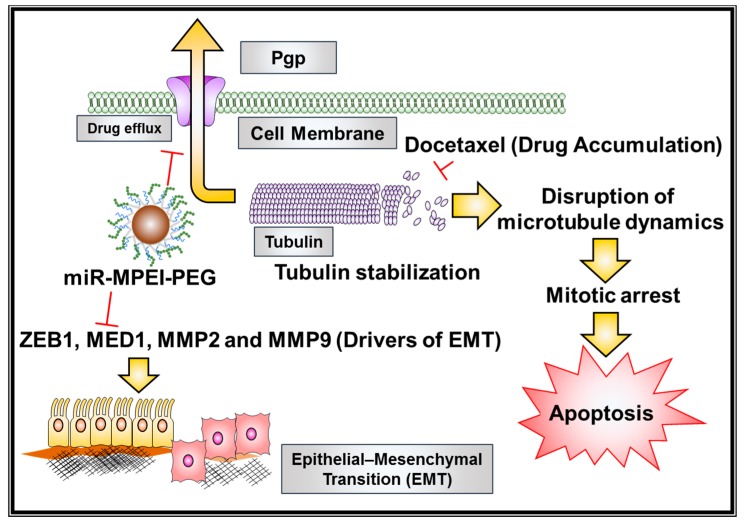
Schematic representation of possible chemosensitization mechanism of action for miR-205-NPs formulation on prostate cancer cells.

The efficacy of docetaxel was measured through the extent of tubulin stabilization during treatments. The percent of tubulin stabilization in cells during drug treatments attests to its level of chemosensitivity towards apoptosis. We found enhanced inhibition of MDR protein after the miR-205 restoration through miR-205-NPs treatment in both C4-2 and PC-3 cells (Figure 7C). In this study, we examine such augmented effects of Dtxl in cells expressing miR-205 with respect to untreated and low miR-205-expressing cells. Morphological images depicted intense green fluorescence in miR-205 cells after Dtxl (5 nM) treatment, demonstrating increased microtubule polymer mass in cells (Figure 7D). The significance of this result indicates enhanced intracellular accumulation of Dtxl, due to inactivity of P-gp (MDR1) miR-205-expressing cells. This explains the enhanced chemosensitive activity of Dtxl in miR-205 cells. Altogether, these results show that miR-205 restoration, after miR-205 NPs treatment in PrCa, enhances chemosensitivity towards docetaxel treatment (Figure 8).

## 3. Discussion

Docetaxel is the most commonly used anticancer drug against advanced metastatic prostate cancer [33]. However, the development of resistance and side effects in patients after Dtxl treatment are common events in clinical use [3]. Some miRNAs function as tumor suppressor genes, and are often downregulated in cancer cells [34]. Among these, miR-205 is a well-known tumor suppressor and is involved in the regulation of cell proliferation, invasiveness, and migration, thus inducing cell death in prostate cancer [7]. miR-205 also plays a significant role in drug resistance [35,36]. Recent literature suggests that overexpression of miR-205 in prostate cancer cells and tumors facilitates a precise and personalized therapeutic option, which can improve treatment and chemosensitization potential [37,38,39]. miR-205 negatively regulates the AR [8], and exerts a tumor suppressive effect [30], also its downregulation interferes with oncogenic pathways [40], tumorigenesis [9], and confers resistance to chemotherapy [10]. Verdoodt et al. [38] confirmed that lower miR-205 expressions in 111 samples of prostate carcinoma is evidence of a significant enlargement of tumor size, and is correlated with an increase of the Gleason score for tumors. Additionally, this study demonstrated that miR-205 inhibits the antiapoptotic protein BCL-xL, affirmed through Western blotting analysis. Another study supports that miR-205 overexpression sensitizes prostate cancer cells for apoptosis by downregulating Bcl-w [10]. Therefore, combined therapy using miR-205 and any chemotherapeutic drug may have the advantage of increased efficacy due to additive anticancer activity.

However, miRNAs are unstable and have a remarkably short half-life due to their degradation during exposure to nucleases in the blood, serum, and other body fluids or tissues [41]. The large molecular size and anionic (negatively charged) surface charge of miRNAs often limits their ability to pass cellular membranes. Therefore, a nonviral delivery using nanoparticles might be a useful strategy for improving miR-205 delivery in PrCa treatment (Figure 1A).

Therefore, our goal was to develop an efficient nanoplatform to deliver miR-205 in its native form to cancer cells (Figure 1, Figure 2 and Figure 3). Poly(ethyleneimine) or cationic-based polymer delivery of miRNAs have inherent higher transfection efficiency via a proton sponge effect at low pH in the endo/lysosomal compartments where it induces membrane-rupture, resulting in the release of miRNAs into the cytoplasm [42]. Our formulation has a slightly positive charge (particle size, ~100 nm), which is highly suitable for miRNAs delivery due to its ability of penetrating tumor sites through leaky blood vessels. This is known as the enhanced permeability and retention effect. This formulation exhibits a significant miR-205 complexation efficiency (Figures 1) and facilitates the process of approaching cell membranes, since it can be internalized through the endocytosis pathway (Figure 3); the complex also avoids endo-/lysosomal degradation, and can thereby deport miR-205 into cytoplasmic compartments (Figure 3). This supports the hypothesis that copolymers of PEI and PEG show better cellular targeting, due to the PEI chains that binds miRNA and deliver it into cells, while the PEG chains minimize aggregation of the nanoparticles. Therefore, this formulation possess favorable attributes for improved reprogramming of miR-205 cellular uptake, which will benefit its induced anticancer and antimetastatic properties, while also reversing the drug resistance phenomenon in prostate cancer cells (Figure 4, Figure 5, Figure 6 and Figure 7).

This nanosized platform composed of an iron oxide core, with branched PEI and PEG grafting, can efficiently deliver the miRNA to the tumor cells. This nanoparticle-mediated delivery of miR-205 induces active antiproliferative, anti-invasive, antimigratory, and chemosensitization functions in prostate cancer cells (Figure 8). We believe such a miR-205 nanoplatform will be a step forward in gene therapy. Overall, this study demonstrates efficient nonviral delivery of miR-205 through the use of novel magnetic nanoparticles, which has built-in drug loading and targeting capacity for future targeted cancer therapeutics.

## 4. Materials and Methods

### 4.1. Materials and Cell Culture

Iron(III) chloride hexahydrate (Fe^3+^ ions) (#236489, 97%, ACS reagent grade), Iron(II) chloride tetrahydrate (Fe^2+^ ions) (#220299, 98% ReagentPlus^®^ ), ammonium hydroxide solution (#320145, 28–30% NH_3_ basis, ACS reagent), branched poly(ethyleneimine) (#408727, MW ~25,000), and dimethyl sulfoxide (#D2650, molecular biology grade) were purchased from Sigma-Aldrich Co. (St. Louis, MO, USA). All other reagents, solvents, chemicals, and cell culture plastics were obtained from Fisher Scientific (Pittsburgh, PA, USA) unless otherwise mentioned. All chemicals were used as received without further modification and purification. All aqueous solutions were prepared using ultrapure water (≥18 MΩ, Milli-Q^®^ Reference Water Purification System, EMD Millipore Corporation, Darmstadt, Germany). Non-targeting control (catalog number AM17111), miR-205 mimics (catalog number 4464066), Taqman miR-205 probes (assay id: 000509), High Capacity cDNA Reverse Transcription Kit (catalog number 4368814, Thermo Fisher Scientific, Grand Island, NY, USA), and TRIzol reagent (catalog number AM 9738) were purchased from Life Technologies (Carlsbad, CA, USA). Prostate cancer cell lines (C4-2 and PC-3) were purchased from American Type Culture Collection (ATCC). These cells were cultured in Roswell Park Memorial Institute-1640 (RPMI-1640) or Dulbecco’s Modified Eagle’s Medium (DMEM) medium (#11875-119, Gibco^®^, Thermo Fisher Scientific, Grand Island, NY, USA) containing 10% (*v*/*v*) fetal bovine serum (FBS) (#10438026, Gibco^®^), and penicillin–streptomycin (10,000 U/mL) (#15140163, Gibco^®^) at 37°C in a humidified 5% CO_2_–95% air chamber (Sanyo scientific Ltd., Tokyo, Japan).

### 4.2. Synthesis of Magnetic Nanoparticles

Iron oxide (Fe_3_O_4_) core was prepared according to the previously described method, i.e., co-precipitation of Fe^3+^ ions and Fe^2+^ ions in the presence of ammonium hydroxide solution under a continuous nitrogen flow [43]. Experimental detail: to 45 mL water in 100 mL beaker, ~810 mg of Fe^3+^ and ~297 mg of Fe^2+^ were added and stirred at 400 rpm on a stir plate (Corning™ Pyroceram™ Top Digital Stirring Hotplates, Fisher Scientific). After 20 min, the stirring speed was increased to 900 rpm and 3 mL of ammonium hydroxide solution was added to form iron oxide core nanoparticles (magnetic nanoparticles). This iron oxide core nanoparticle generation was an instantaneous process and excess ammonia was evaporated by heating the stir plate to 60 °C for 30 min. Then this suspension was allowed to stir overnight at 25 °C with PEI-PEG [conjugated 1:1 ratio of PEI (#764582, Aldrich, St. Louis, MO, USA) and PEG (PG2-NS-5k, NANOCS, New York, NY, USA)] at 400 rpm. During this step, the PEI-PEG coating was achieved. At the end of the reaction time, and after three washes with water, the nanoparticles were resuspended and larger aggregates were removed with centrifugation at 1000 rpm (Sorvall ST8; Thermo Scientific, Waltham, MA, USA). The supernatant containing iron oxide core nanoparticles (MNPs), and iron oxide core nanoparticles coated with PEI-PEG (MNP-PEI-PEG or MPEI-PEG or NPs for convenient representation) were stored as stock solutions until further use.

### 4.3. Particle Characterization

Particle size, distribution, and zeta potential measurements of nanoformulations were determined by dynamic light scanning analysis employing a Malvern Zetasizer (nano ZS, Malvern, Westborough, MA, USA). For this study, diluted nanoparticle suspensions (1 mg/mL) were probe sonicated using a VirSonic Ultrasonic Cell Disrupter 100 (VirTis, Gardiner, NY, USA) for 30 s and measurements performed at room temperature [44,45,46]. The mean hydrodynamic diameter was determined via cumulative analysis of three runs for each formulation for 3 min. The zeta potential was acquired using the principle of electrophoretic mobility under an electric field. The value reported was an average of 3 measurements (~9 min). The sizes and morphologies of nanoformulations were determined by JEOL 200EX transmission electron microscopy (TEM) (JEOL Ltd., Tokyo, Japan) operating at 60 kV. A nanoparticle suspension (100 μg/mL) was prepared and probe sonicated for 45 s [47]. Two hundred mesh Formvar-coated copper TEM grid (grid size 97 μm; Ted Pella Inc., Redding, CA, USA) was employed in sample preparation, and the nanoparticle suspension (20 μL) was carefully placed on the darker lateral of the grid. The excess amount of formulation suspension on the grid was removed by filter paper and air dried followed by imaging.

### 4.4. miR-205 Binding with Nanoparticles

The complexation or binding profile of miR-205 mimic (Mature miRNA Sequence: UCCUUCAUUCCACCGGAGUCUG, Thermo Fisher, referred to as miR-205 throughout this work) with developed nanoparticles (MNPs and MPEI-PEG NPs) was evaluated using gel retardation, fluorescence quenching, and circular dichroism assays. For the gel retardation analysis, the complexes were prepared by adding 5 µg miR-205 to 1–10 µg nanoparticles, vortexed immediately, and allowed to stand 30 min. The total binding reaction was conducted in 20 µL of solution. The complexed solutions were subjected to gel electrophoresis using 2% agarose gel in presence of agarose gel ladder [48]. Then, the gels were stained with 0.5 µg/mL ethidium bromide for 20 min and analyzed on a UV illuminator (UVPTM Multi-User Imaging System, Upland, CA, USA) to identify the mobility or location of the miRNA. In the fluorescence quenching study, 10 µg/mL FAM-labelled miRNA mimic (AM17121, FAM3™ Dye-Labeled Pre-miR Negative Control) solution was titrated against MNPs or MPEI-PEG (2.5–50 µg) to determine the binding between microRNA and nanoparticles by measurement of quenching through fluorescence reduction (SpectraMax Plus Plate Reader, Molecular Devices, Sunnyvale, CA, USA). Additionally, the binding confirmation of miR-205 (100 µg/mL) with MPEI-PEG (50 µg/mL) was determined by recording secondary structures of miR-205 using Aviv 410 CD Spectrometer (Lakewood, NJ, USA). Spectra were recorded between 350 nm and 190 nm wavelength at a speed of 1 nm bandwidth/1 nm wavelength step at 25 °C.

### 4.5. Hemolytic Assay

The hemolytic assay was conducted to determine compatibility and safety of MPEI-PEG nanoformulation by measuring the lysis of red blood cells (RBCs). In brief, the RBCs were isolated from 1 mL of a healthy male whole blood (Interstate Blood Bank, Inc., Memphis, TN, USA). The RBCs diluted to 8 mL PBS solution. Various concentrations MPEI-PEG NPs were incubated in 200 µL of RBCs suspension solution for 2 h. Hemolysis was determined from the absorbance measured at 570 nm using Cytation™ 5 (BioTek Instruments, Inc., Winooski, VT, USA). The amount of hemoglobin released in the presence of SDS was considered as 100% lysis (Positive control). The percent hemolysis was calculated as: [(Abs. of formulation − Abs. of SDS sample)/(Abs. of PBS sample − Abs. of SDS sample)] × 100. Aliquots of above treated RBCs were mounted on a glass slide (Fisher scientific, Waltham, MA, USA) and morphology of RBCs acquired using a microscope (EVOS FL Cell Imaging System, Carlsbad, CA, USA).

### 4.6. Stability of microRNA

For the NPs (MPEI-PEG), 1 µg of miR-205 was incubated for dissociation using heparin sulfate at varying concentrations for 45 min to ensure complete release of miRNA from the formulations and run on 2% agarose gel [49]. The miRNA bands were digitized and quantified using ImageJ analysis software to determine the mean density of bands.

### 4.7. miRNA Stability in Serum

To determine the stability of miR-205 against serum degradation in the NPs, the formulation was prepared and incubated with 25% fetal bovine serum for 24 h at 37 °C followed by dissociation using heparin sulfate for 45 min to ensure complete release of miRNA from the formulations into suspension, and run on agarose gel. miRNA bands were digitized and quantified using Image analysis software to determine the mean density of the miRNA bands [49].

### 4.8. miRNA Release Assay

The extent of miRNA release from the nanoparticle formulation was evaluated using a variable pH microenvironment. In this study, we assessed the miRNA dissociation ability of MPEI-PEG formulation at variable pH: 3.5 (acidic), 6.5 (tumor microenvironment), and 7.4 (physiological pH) in PBS, with slight modifications [50]. For this assay, we encapsulated FAM miRNA mimic within MPEI-PEG, and the resultant formulation was subjected to variable pH and for different time intervals like 0, 0.5, 3, 6 and 24 h. After incubation, the nanoparticle mixture was centrifuged at 3000 rpm for 5 min and the respective supernatant was collected and measured for fluorescence intensity using a microplate reader at 488 nm.

### 4.9. Cellular Uptake and Transfection Efficiency

The uptake phenomenon of nanoparticles in cells is a primary indication for a possible route of delivery for loaded therapeutics. For this analysis, MPEI-PEG nanoformulation was tagged with coumarin 6 following our previous protocol (~100 µg of coumarin 6/mg of nanoparticles). The uptake study was conducted in an independent method of analysis using flow cytometry [21,51] and confocal microscopy [44,51]. For flow cytometry cellular uptake analysis, C4-2 and PC-3 prostate cancer cells (2 × 10^4^/well in 6-well pate) were incubated with 5, 10, 15, and 20 µg dye equivalent MPEI-PEG nanoformulation. After 2 h, cells were washed with cold-PBS solution, trypsinized, collected in phenol-free medium, and the extent of uptake of nanoparticles was detected using Accuri C6 Flow Cytometer (BD Biosciences, San Jose, CA, USA). Green fluorescence from coumarin 6 in nanoparticles was measured in the FL1 channel (488 excitation, blue laser, 530 ± 15 nm, FITC/GFP). In the confocal uptake study, C4-2 and PC-3 cells (1 × 10^5^/well in 4-well chamber slides) (Sarstedt., Newton, NC, USA) were incubated with 2.5, 5, and 10 μg of coumarin 6 equivalent MPEI-PEG for 2 h. Cells were permeabilized with 0.1% TritonX-100, washed twice with PBS solution, stained with 4′,6-diamidino-2-phenylindole (DAPI, Life Technologies). Chamber slides were mounted in Vectashield Mounting Medium (Vector Labs, Burlingame, CA, USA) with coverslip. Then, the uptake of nanoparticles was visualized using a laser confocal microscope (Carl Zeiss LSM 710, Oberkochen, Germany) at excitation/emission wavelengths of 360/460 nm (DAPI, blue) and 488/518 nm (coumarin 6, green). All images were taken at 400× magnification.

The transfection efficiency of miRNA in cells was evaluated using flow cytometry. In this study, the mean fluorescence intensity (MFI) of FAM, a chromogen-labelled miRNA (Thermo Fisher Inc., Waltham, MA, USA), was evaluated. PrCa cells (2.5 × 10^5^) were transfected/treated with FAM-miRNA through lipofectamine (conventional) and NPs formulation. After 24 h of incubation, cells were trypsinized and suspended in phenol red-free medium and followed by a flow run of the sample. Finally, the MFI values from the flow cytometer were tabulated and graphed.

### 4.10. Transfection and Treatments

In all of the transection studies, miR-205 (mimic) and negative control mimics (Carlsbad, CA, USA) (partially double-stranded RNAs that mimic the Dicer cleavage product and are subsequently processed into their respective mature miRNAs) (Lafayette, CO, USA) were used. Once the C4-2 or PC-3 cells reached confluency (6 well plate) of 80%, transfection of the cells was facilitated with 5 µg of miR-205 or control mimics with Lipofectamine 3000 reagent (Invitrogen, Carlsbad, CA, USA) per the manufacturer’s instructions [52]. Similarly, 5 µg of miR-205 loaded with NPs formulation (miR-205-NPs) was employed in a transfection experiment. but with no transfection reagents added. After transfection, cells were used for all in vitro studies (proliferation-MTS, colony formation, Western blotting, and qRT-PCR analysis) with or without Dtxl treatment for evaluating the chemosensitization potential of miR-205NPs formulation.

### 4.11. Anticancer Efficacy of miR-205

#### 4.11.1. MTS Assay

The anticancer efficacy of miR-205 was determined by MTS and colony formation assays. Briefly, miR-205/miR-205-NPs-transfected C4-2 and PC-3 cells were seeded at a cell density of 5 × 10^3^ cells per well in a 96-well plate. These cells were treated with 1.5–25 nM Dtxl, or were untreated, for 48 h. Then, cells were incubated with 20 µL MTS reagents for 2 h, and the absorbance was measured at 592 nm using a plate reader (Cytation 5, Biotek Instruments, Winooski, VT, USA) [51]. Cells without miR-205/therapeutic treatment served as control. The percent cell viability of treated samples following incubation was calculated with respect to the above control according to our protocol.

#### 4.11.2. Colony Formation Assay

In a colony formation assay, transfected cells were seeded on 6-well plates at a cell density of 500 cells/well. After day 2, these cells were treated with 2.5 nM Dtxl or were untreated. After 7 days of treatment (or cultured without being treated), the culture medium was changed but no therapeutic agents were added. On day 14, the grown colonies were fixed, stained, and imaged using Nikon Eclipse microscope (Nikon Instruments Inc., Melville, NY, USA) [53].

#### 4.11.3. Cell Migration and Invasion Assays

Cell migration was analyzed using a Boyden chamber assay, as described previously [54,55]. Briefly, following transfection with NC, miR-205-Lipo and miR-205-NPs, in the presence of prostate cancer cells C4-2 and PC-3, the cells were plated (5 × 10^5^ cells/well) to form a monolayer. Further, these transfected cells were exposed to Dtxl treatments for assessing the chemosensitivity effects in miR-205-expressing cells. The cell invasion assay was performed to investigate the effect of miR-205-NPs on the cells using BD Biocoat Matrigel Invasion Chambers (BD Biosciences), per manufacturer’s protocol [22,56]. After 48 h incubation, the invading cells were fixed with methanol and stained with crystal violet. The invading cells were counted and plotted as percent invasion of the miR-205-NPs-treated cells compared to control (NC).

### 4.12. Western Blotting

After transfecting cells with miR-205-Lipo or miR-205-NPs, cells were treated both in the presence/absence of 5 nM Dtxl for 48 h. Cells were washed with ice-cold PBS, then harvested in CelLytic M Cell Lysis Reagent (Sigma Aldrich, St. Louis, MO, USA) containing protease and phosphatase inhibitors (Thermo Fisher, Waltham, MA, USA). Protein quantity was determined using a BCA protein assay kit (Thermo Fisher, Waltham, MA, USA) [57]. Equal amounts of protein and cell lysates were loaded into 4–20% polyacrylamide gel, and proteins were then separated using SDS-PAGE, and transferred electrophoretically onto nitrocellulose membranes, followed by a blocking step, as described in our previous report [57]. Western blots were probed with primary antibodies such as, ZEB1 (Thermo Fisher, Waltham, MA, USA, #PA5-20979), MED1 (Thermo Fisher, #PA5-36200), MMP9 (#3852), MMP2 (#4022), vimentin (#5741), slug (#9585), cleaved PARP (#9548), Bcl-xL (#2764), cleaved caspase 3(#9665), Bid (#2002), and secondary antibody (horseradish peroxidase-conjugated goat anti-mouse or goat anti-rabbit secondary antibody) (Cell signaling, Danvers, MA, USA). The bands were imaged with a Bio-Rad computer-based gel imaging instrument and analyzed using ImageLabTM software (5.2.1, Bio-Rad, Hercules, CA, USA) and band intensities analyzed employing the ImageJ program (1.50b, NIH, Bethesda, MD, USA.) software.

### 4.13. Rhodamine 123 Uptake/Retention Assays

For this study, transfected prostate cancer cells (2.5 × 10^5^ cells/well) were seeded and treated with 5 nM Dtxl for 24 h. These cells were processed for Rh123 uptake studies. Cells were stained with Rh123 (500 ng/mL of the dye, stock solution 1 mg/mL in distilled water) added to the culture medium [58]. Then cells were trypsinized and processed for the evaluation of Rh123 dye retention ability in cells. The cellular efflux of Rh123 was measured by monitoring its fluorescence decrease at 525 nm emission wavelength. The analysis of 1 × 10^4^ cells per sample was carried out in the Rh123/count for fluorescence signal. All analyses were performed in triplicate in three separate experiments, and the results were expressed as the mean of fluorescence intensity. In another set of experiments, after treatment and addition of Rh123 dye, the culture medium was replaced with phenol red-free medium and cells were imaged using an epifluorescence imaging system from Advanced Microscopy Group EVOS^®^ microscopic system (Mill Creek, WA, USA).

### 4.14. Tubulin Stabilization Assay

The influence of Dtxl treatment on tubulin stabilization in cells transfected with miR-205 and miR-205-NPs was studied using immunofluorescence assay [43]. For this measurement, prostate cancer cells (2.5 × 10^4^ cells/mL in each slide) were seeded in chambered slides and grown for 24 h. Cells were treated with 5 nM Dtxl for 8 h with no treatment in controls. After incubation, cells were washed with PBS, fixed with ice-cold methanol for 20 min, blocked with 10% goat serum for 1 h, and incubated overnight with β-tubulin antibody (1:50 Santa Cruz, #31782) at 4 °C. After washing, cells were then probed with FITC-conjugated goat anti-rabbit secondary antibody (1:200, #7074, Cell Signaling, Danvers, MA, USA) for 1 h, and the nuclei were counterstained with DAPI mounting medium and visualized under a laser confocal microscopy (Carl Zeiss LSM 710).

### 4.15. Statistical Analysis

All statistical analyses were performed using GraphPad Prism (5.03, GraphPad Software, Inc., La Jolla, CA, USA). Biological assay data were presented as mean ± SEM of at least three sets of experiments. A *p* value ≤ 0.05 was considered statistically significant.

## 5. Conclusions

We have developed a magnetic nanoparticle-based miR-205-NPs delivery platform composed of iron oxide core with PEI-PEG coating. The obtained formulation had particle size and zeta potential that were in the therapeutic formulation range. This platform demonstrated no signs of hemotoxicity in preliminary safety assessments on human red blood cells. The miR-205 nanoplatform demonstrates superior transfection efficiency, superior anticancer properties, and chemosensitization effects in prostate cancer cells. Overall, these results provide a solid groundwork for future opportunities to develop functional miR-205-NPs nanoformulation-based treatment strategy for prostate cancer.

## Supplementary Materials

The following are available online at http://www.mdpi.com/2072-6694/10/9/289/s1, Figure S1: DLS characterization of miR-205-NPs. (A) Particle size of MPEI-PEG and miR-205-NPs. (B) Zeta potential of MPEI-PEG and miR-205-NPs. (C) Transmission electron micrographs of MPEI-PEG and miR-205-NPs formulations; Figure S2: Cellular uptake studies of miR-205-NPs. Cellular uptake of coumarin 6-labeled MPEI-PEG NPs formulation. The level of significance was * *p* < 0.05. Each individual experiment has been repeated three times; Reverse transcription–quantitative real-time PCR (qRT-PCR) protocol; Figure S3: miR-205-NPs treatment chemosensitizes PrCa cells towards docetaxel therapy. NC, NPs, miR-205-Lipo, and miR-205-NPs-treated PrCa cells with 2.5 nM Dtxl. On day 14, cells were PBS-rinsed and stained with hematoxylin. Colonies were counted using Multimage™ light cabinet. Clonogenic ability in (A) C4-2 and (B) PC-3 PrCa cells. The level of significance is represented as * *p* < 0.05; Figure S4: Gene regulatory effects of miR-205-NPs in PrCa cells. (A,B) mRNA expression levels of E-Cadherin, MMP9 and MMP2 (EMT signaling) proteins in miR-205-NPs plus Dtxl treated cells (through qPCR studies) in C4-2 and PC-3 cells. The level of significance was * *p* < 0.05. Each individual experiment has been repeated three times; Table S1: Primer sequence of miR-205 and its downstream-related proteins.
