# Supplementary material for: miRNA-205 Nanoformulation Sensitizes Prostate Cancer Cells to Chemotherapy
Source: Cancers (Basel). 2018 Aug 25;10(9):289. doi: 10.3390/cancers10090289 (PMC6162422; doi:10.3390/cancers10090289)
Supplement: Supplementary file 1 [file cancers-10-00289-s001.pdf]

Supplementary Materials: miRNA-205 Nanoformulation Sensitizes Prostate Cancer Cells to Chemotherapy

Prashanth K.B. Nagesh, Pallabita Chowdhury, Elham Hatami, Vijaya K.N. Boya, Vivek K. Kashyap, Sheema Khan, Bilal Bin Hafeez, Subhash C. Chauhan, Meena Jaggi and Murali M. Yallapu

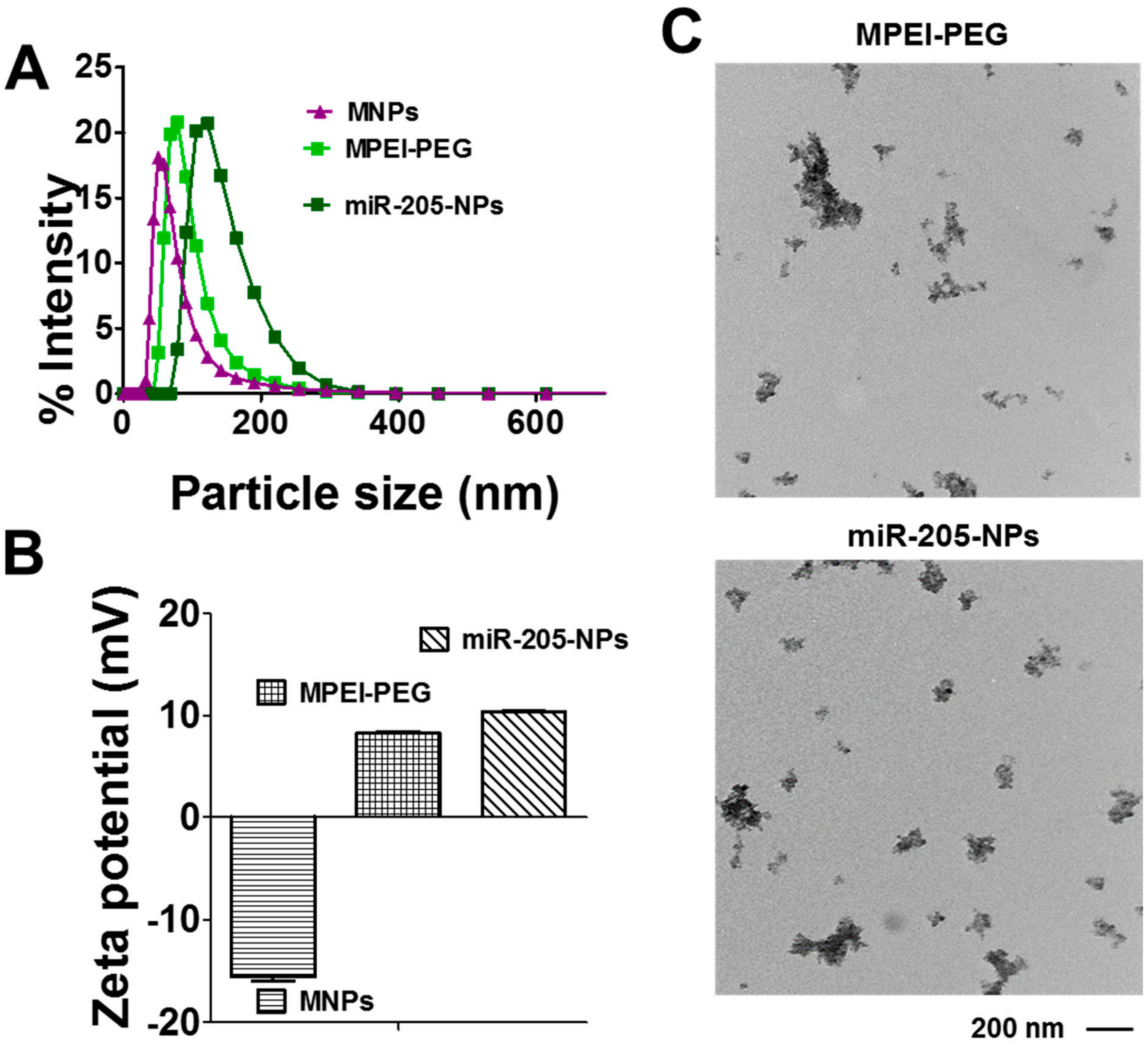

Figure S1. DLS characterization of miR-205-NPs. (**A**) Particle size of MPEI-PEG and miR-205-NPs. (**B**) Zeta potential of MPEI-PEG and miR-205-NPs. (**C**) Transmission electron micrographs of MPEI-PEG and miR-205-NPs formulations.

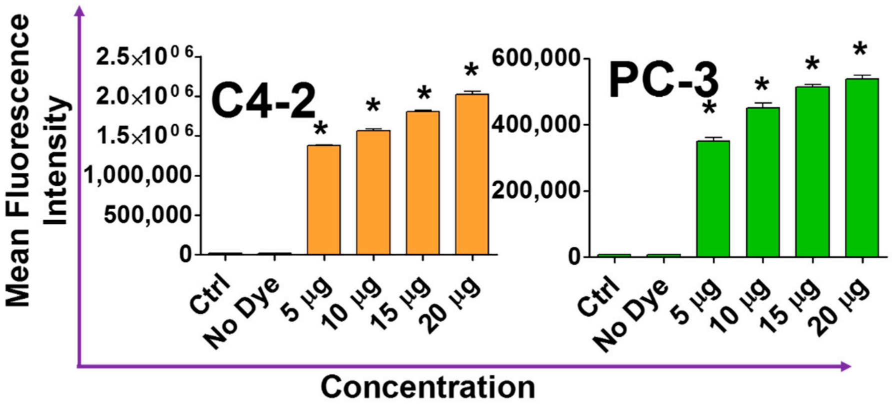

Figure S2. Cellular uptake studies of miR-205-NPs. Cellular uptake of coumarin 6 labeled MPEI-PEG NPs formulation. The level of significance is represented as * $p < 0.05$. Each individual experiment has been repeated three times.

Reverse transcription–quantitative real-time PCR (qRT-PCR)

Cells were collected from above treatment method and total RNA was extracted from the treated cells using TRIzol reagent (Invitrogen, Life Technologies, and Grand Island, NY) and chloroform, followed by a purification with isopropanol precipitation and washed with 70% ethanol [1, 2]. The integrity of the RNA was checked with an RNA 6000 Nano Assay kit and 2100 Bioanalyzer (Agilent Technologies, Santa Clara, CA). For miRNA detection, 100 ng total RNA was reverse transcribed into cDNA using specific primers designed for miRNA analysis (Applied Biosystems, Foster City, CA). The mRNA expression of genes were analyzed by qPCR using specific primers sequences as described [3] in supplementary Table S1. Expression of miR-205 was determined by qPCR using the Taqman PCR master mixture (no AmpErase UNG) and specific primers designed for detection of mature miRNAs (Applied Biosystems). The expression of miRNA was normalized with U6snRNA (endogenous control). Relative gene expression values were determined by the $\Delta\Delta C_t$ method. The expression of E-Cadherin, MMP-2, and MMP-9 was normalized to GAPDH, which was the endogenous reference in the corresponding samples, and relative to the untreated control cells. The primers for this assay was designed considering a previous report [4].

Table S1. Primer sequence of miR 205 and its downstream related proteins.

Primer Name	Sequence
MIR205F	GCGGCGGTGTAGTGTTTCCTA
MIR205R	GTGCAGGGTCCGAGGT
MMP-2 F	TCTCCTGACATTGACCTTGGC
MMP-2 R	CAAGGTGCTGGCTGAGTAGATC
MMP-9 F	TTGACAGCGACAAGAAGTGG
MMP-9 R	GCCATTCACGTCGTCCTTAT

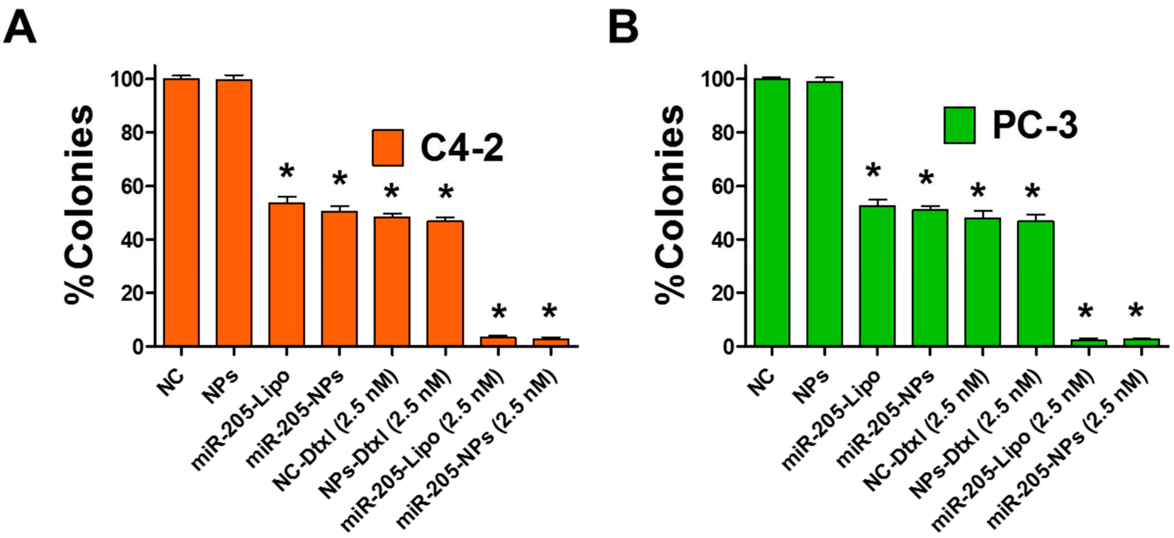

Figure S3. miR-205-NPs treatment chemosensitizes PrCa cells towards docetaxel therapy. NC, NPs, miR-205-Lipo, and miR-205-NPs-treated PrCa cells with 2.5 nM Dtxl. On day 14, cells were PBS-rinsed and stained with hematoxylin. Colonies were counted using Multimage™ light cabinet. Clonogenic ability in (**A**) C4-2 and (**B**) PC-3 PrCa cells. The level of significance is represented as * $p < 0.05$.

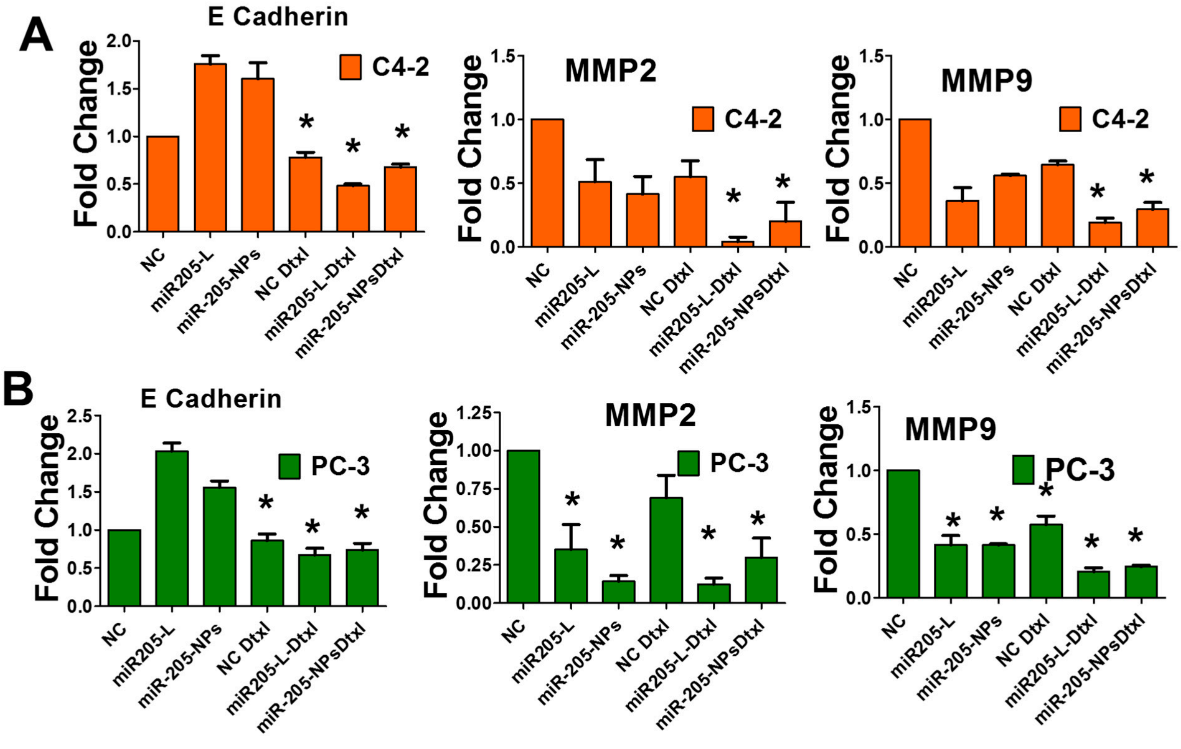

Figure S4. Gene regulatory effects of miR-205-NPs in PrCa cells. mRNA expression levels of E-Cadherin, MMP9 and MMP2 (EMT signaling) proteins in miR-205-NPs plus Dtxl treated cells

(through qPCR studies) in **(A)** C4-2 and **(B)** PC-3 PrCa cells. The level of significance is represented as * $p < 0.05$. Each individual experiment has been repeated three times.

References

1. Sikander, M.; Hafeez, B.B.; Malik, S.; Alsayari, A.; Halaweish, F.T.; Yallapu, M.M.; Chauhan, S.C.; Jaggi, M. Cucurbitacin d exhibits potent anti-cancer activity in cervical cancer. *Sci. Rep.* **2016**, *6*, 36594.
2. Dey, K.K.; Pal, I.; Bharti, R.; Dey, G.; Kumar, B.N.P.; Rajput, S.; Parekh, A.; Parida, S.; Halder, P.; Kulavi, I.; et al. Identification of rab2a and prdx1 as the potential biomarkers for oral squamous cell carcinoma using mass spectrometry-based comparative proteomic approach. *Tumor Biol.* **2015**, *36*, 9829-9837.
3. Lobert, S.; Hiser, L.; Correia, J.J. Expression profiling of tubulin isotypes and microtubule-interacting proteins using real-time polymerase chain reaction. *Methods Cell Biol.* **2010**, *95*, 47-58.
4. Nagesh, P.K.B.; Johnson, N.R.; Boya, V.K.N.; Chowdhury, P.; Othman, S.F.; Khalilzad-Sharghi, V.; Hafeez, B.B.; Ganju, A.; Khan, S.; Behrman, S.W.; et al. Psma targeted docetaxel-loaded superparamagnetic iron oxide nanoparticles for prostate cancer. *Colloids Surf. B Biointerfaces* **2016**, *144*, 8-20.
